# Moiré-Based Alignment Using Centrosymmetric Grating Marks for High-Precision Wafer Bonding

**DOI:** 10.3390/mi10050339

**Published:** 2019-05-22

**Authors:** Boyan Huang, Chenxi Wang, Hui Fang, Shicheng Zhou, Tadatomo Suga

**Affiliations:** 1 School of Instrumentation Science and Engineering, Harbin Institute of Technology, Harbin 150001, China; byhuang@hit.edu.cn; 2 State Key Laboratory of Advanced Welding and Joining, Harbin Institute of Technology, Harbin 150001, China; hitfanghui@163.com (H.F.); zhoushichengwow@163.com (S.Z.); 3 Department of Precision Engineering, School of Engineering, The University of Tokyo, Bunkyo, Tokyo 113-8656, Japan; suga@gakushikai.jp

**Keywords:** moiré fringe, centrosymmetric grating, measurement range, alignment mark, wafer bonding, alignment accuracy

## Abstract

High-precision aligned wafer bonding is essential to heterogeneous integration, with the device dimension reduced continuously. To get the alignment more accurately and conveniently, we propose a moiré-based alignment method using centrosymmetric grating marks. This method enables both coarse and fine alignment steps without requiring additional conventional cross-and-box alignment marks. Combined with an aligned wafer bonding system, alignment accuracy better than 300 nm (3σ) was achieved after bonding. Furthermore, the working principle of the moiré-based alignment for the backside alignment system was proposed to overcome the difficulty in bonding of opaque wafers. We believe this higher alignment accuracy is feasible to satisfy more demanding requirements in wafer-level stacking technologies.

## 1. Introduction

Wafer bonding is regarded as one of the most promising technologies for three-dimensional (3D) heterogeneous integration [1,2,3,4]. Wafer-to-wafer alignment accuracy specifications are more demanding due to smaller features in the fabrication of nanoelectronics and nanosystems [5,6,7]. For aligned wafer bonding, conventional alignment marks (e.g., “cross-and-box” or “cross-on-cross” structures) are pre-fabricated in the top and bottom wafers. Optical or infrared (IR) microscopes are used to detect the alignment marks. Once the mark images are captured, the relative position of the top and bottom marks can be acquired with a peak-detection algorithm. The alignment technique is simple in principle, but its accuracy is generally limited by the resolution of the microscope. Complex sub-pixel peak estimation algorithms were thus employed in the image detection process. A variety of wafer alignment tools were developed for high-precision wafer bonding, in which the alignment accuracy was improved to sub-μm range, even using conventional alignment marks [8,9]. However, the accuracy is difficult to improve continuously by optimizing the mark detection alone. On the other hand, moiré fringes generated by the overlaying of two sets of gratings with slightly different periods have extremely high alignment sensitivity more than just optics [10,11]. Therefore, a variety of moiré fringe measurement methods were developed for alignment in proximity optical, x-ray, and nanoimprint lithography [12,13,14].

In recent years, we demonstrated a moiré fringe assisted alignment method using centrosymmetric gratings, which exhibited many advantages over conventional moiré gratings [15,16]. For instance, small planar misalignments and misaligned directions can be easily measured without requiring any external reference. However, the measurement range is small (just a quarter of the grating pitch, around several microns in most applications) [17]. The coarse alignment must be carried out using conventional cross-and-box marks before the moiré-based fine alignment. Thus, additional alignment marks are still necessary for the moiré fringe assist alignment method. In this case, two measurement systems have to be adopted based on the image detection method and the grating interference method separately. Moreover, the two kinds of alignment marks will occupy larger areas in each wafer, leading to additional cost and complexity. It is highly desirable to explore a solution that combines coarse and fine alignment process with one kind of the alignment mark.

In this paper, the moiré patterns with small and large misalignments are investigated separately. We propose a moiré-based alignment method using centrosymmetric grating images, which enables both coarse and fine alignment steps without the assistance of additional alignment marks. This method was applied to the aligned wafer bonding system. Consequently, we tested the moiré-based alignment accuracy after the bonding.

## 2. Principle and Methods 

**Figure 1:**
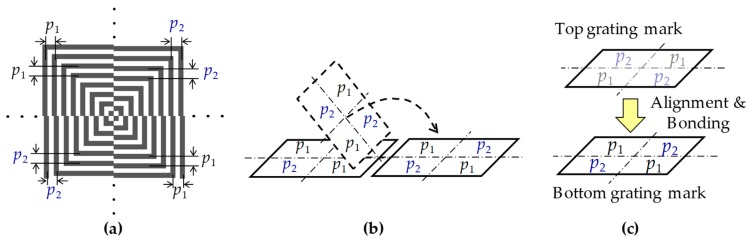
(**a**) Layout of the centrosymmetric grating mark for moiré-based alignment, (**b**) the top grating mark turns over and are superimposed onto the identical bottom grating mark, the moiré fringes are therefore formed during alignment and bonding processes in (**c**).

The layout of the centrosymmetric grating mark for the moiré-based alignment is illustrated in Figure 1a. The mark consists of four sets of L-shaped gratings. The gratings of the first and the third quadrants have the pitch of *p*_1_; while the gratings of the second and the fourth quadrants have the pitch of *p*_2_, which is equal to *p*_1_ plus an additional term Δ*p* (> 0), that is, *p*_2_ = *p*_1_ + Δ*p*. There is no difference between the top and bottom grating marks. It is convenient for the mark design and fabrication. When the top grating mark turns over, the gratings with different pitches are superimposed (Figure 1b). Therefore, the moiré fringes can be formed during alignment and bonding processes, as shown in Figure 1c. Here, all of the moiré fringes have the same period *P*, expressed as

(1) $$P=\frac{p_{1}p_{2}}{p_{2}-p_{1}}=\frac{p_{1}p_{2}}{\Delta p}$$

**Figure 2:**
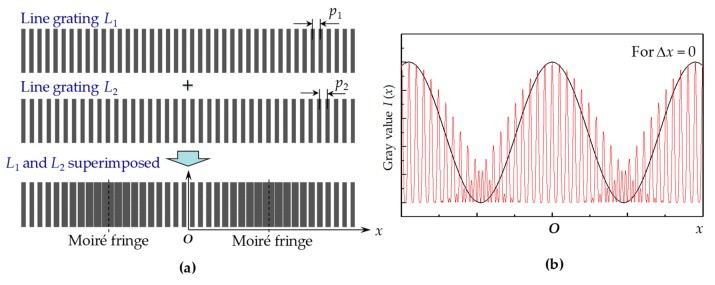
(**a**) Moiré fringes generated by the superimposing two line gratings (*L*_1_ and *L*_2_) with slightly different pitches (*p*_1_ and *p*_2_); (**b**) the intensity distribution (red line) of superimposed grating image along *x*-direction, from which a sinusoidal envelop curve (black line) can be derived to address the peaks of moiré fringes more clearly.

The methodology of the digital moiré fringes is explained in Figure 2. Moiré fringes are generated by superimposing two line gratings (*L*_1_ and *L*_2_) with slightly different pitches (*p*_1_ and *p*_2_). In general, the grating images can be captured by charge-coupled device (CCD) cameras or optical microscopes. When the light beam transmits through the line gratings, the intensity distribution of the line gratings along the *x* axis direction can be assumed sinusoidal as

(2) $$I_{i}(x)\;=\;\frac{1}{2}I_{0}\left(1+\cos\frac{2\pi x_{i}}{p_{i}}\right)\;i=1,2$$

where *I*_0_ is the maximum gray level of grating image, *p_i_* is the line pitch (*i* = 1 or 2), *x_i_* represents the *x* coordinate of the grating *L*_1_ or *L*_2_. When two grating images are superimposed, the intensity distribution of superimposed grating image along *x*-direction is described by the following equation.

(3) $$I(x)=I_{1}(x)\times I_{2}(x)$$

There are five terms in the expansion Equation (3), of which only the low spatial frequency term contributes to the moiré fringes. After unwanted frequency terms were filtered, a sinusoidal envelop curve (black line) could be derived to address the peaks of moiré fringes more clearly, as shown in Figure 2b. 

**Figure 3:**
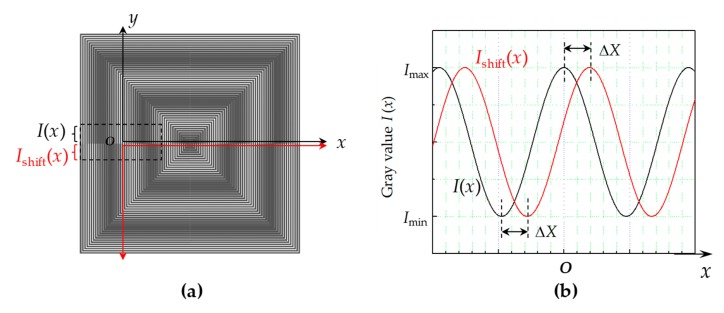
(**a**) Moiré fringe pattern with a small misalignment by Δ*x* (≠0), which will lead to a moiré shift (Δ*X*) in the *x*-direction between the two rows of moiré fringes; (**b**) the moiré shift can be determined by detecting the phase difference between two digital moiré fringes, i.e., *I*(*x*) and *I*_shift_(*x*).

The magnification effect of the moiré fringe allowed the manifestation of a small displacement (i.e., Δ*x*) in a large moiré fringe shift (i.e., Δ*X*). For the centrosymmetric moiré gratings, two rows of moiré fringes moved opposite during the linear displacement along *x*-direction, as shown in Figure 3a. This doubled the measurement sensitivity. The intensity distribution can be expressed approximately by the following equation, and is shown in Figure 3b.

(4) $$I(x)\;=\;I_{0}\cos\left[2\pi \left(\frac{1}{p_{2}}-\frac{1}{p_{1}}\right)x\;-2\pi \left(\frac{1}{p_{2}}+\frac{1}{p_{1}}\right)\Delta x\right]$$

The moiré shift can be determined by detecting the phase difference between two digital moiré fringes, that is, *I*(*x*) and *I*_shift_(*x*). According to Equation (4), the actual displacement in *x* or *y* directions can be magnified by a factor *M*

(5) $$M\;=\;\frac{\Delta X}{\Delta x}=\frac{\Delta Y}{\Delta y}=\frac{p_{1}+p_{2}}{p_{2}-p_{1}}=\frac{p_{1}+p_{2}}{\Delta p}$$

**Figure 4:**
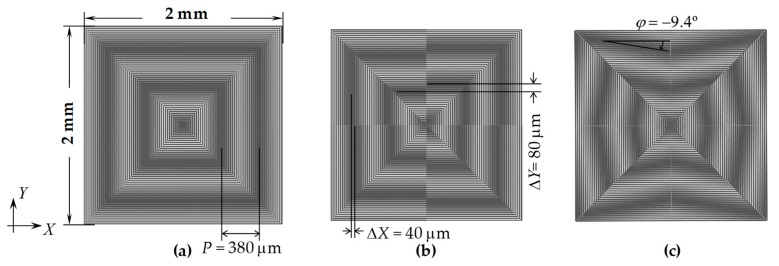
Moiré fringe patterns with various misalignments as examples: (**a**) Δ*x* = Δ*y* = 0; (**b**) Δ*x* = 1 μm, Δ*y* = 2 μm; (**c**) Δ*x* = Δ*y* = 0 with a small angular error (*δ* = 0.5°).

The relationships between the misalignments and moiré fringes were investigated. In our case, *p*_1_ = 19 μm and *p*_2_ = 20 μm, the period of moiré fringe is *P* = 380 μm and *M* ≈ 40, hereinafter. Figure 4 shows moiré fringe patterns with small misalignments. In Figure 4a, the two grating marks are superimposed with no misalignment, that is, Δ*x* = Δ*y* = 0. When the two gratings were misaligned by Δ*x* = 1 μm and Δ*y* = 2 μm, one moiré fringe evolved into two mismatched moiré fringes (Figure 4b), whose distances were magnified by the factor of *M* according to Equation (5). The distances between the moiré fringes corresponded to Δ*X* = 40 μm and Δ*Y* = 80 μm, which can be easily detected and measured without requiring any external reference. In addition to linear misalignments, when there was a small angular error between the two grating marks, the magnification of the grating image generated an angular moiré fringe, as shown in Figure 4c. With a small angular error of *δ*, the digital moiré fringes have a large angular inclination *φ*

(6) $$\varphi \;=\;\tan^{-1}\frac{p_{1}\sin\delta }{p_{1}\cos\delta -p_{2}}\;$$

**Figure 5:**
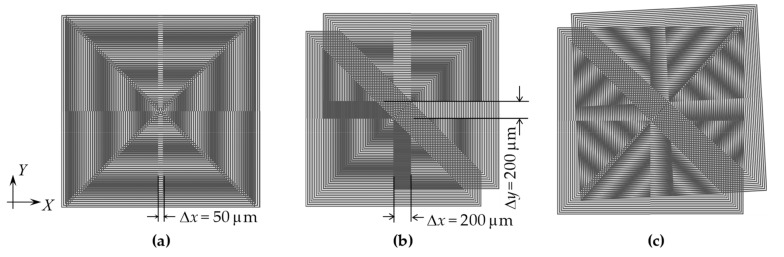
Moiré fringe patterns with large misalignments beyond the moiré measurement range: (**a**) Δ*x* = 50 μm, Δ*y* = 0; (**b**) Δ*x* = Δ*y* = 200 μm; (**c**) Δ*x* = Δ*y* = 200 μm with an angular error (*δ* = 3°).

Figure 5 shows moiré fringe patterns with large misalignments. The two gratings were interlaced together at the central region without the moiré fringes because the superimposed gratings have the same pitch (Figure 5a). When there were large misalignments in *x* and *y* directions between the two gratings, a crossbar-like image appeared in the central region (Figure 5b). The peak-detection algorithm is particularly appropriate for the crossbar-like image at the central region without moiré fringe interference. Moreover, the angular errors often occurred together with large linear misalignments when the two wafers were initially aligned, resulting in large inclinations of the moiré fringes, as shown in Figure 5c. Thus, the large linear misalignments and the angular errors could be easily detected with image processing. Hence, the coarse alignment could be carried out using these square centrosymmetric gratings without requiring conventional alignment marks.

## 3. Experiment and Results

**Figure 6:**
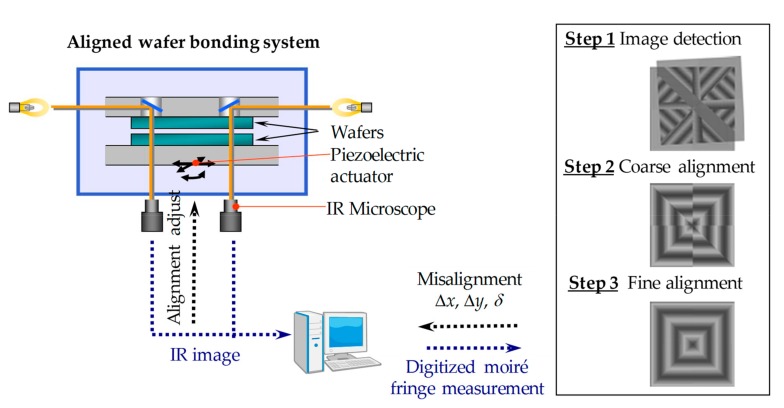
Schematic illustration of the aligned wafer bonding system and the moiré-based alignment process using centrosymmetric grating marks.

Figure 6 shows an aligned wafer bonding system and the moiré-based alignment process using centrosymmetric grating marks. The alignment and bonding chamber is the main part of the wafer bonding system, which consists of a wafer transfer pin lift shaft, electrostatic chucks holding the top and bottom wafers, a piezoelectric walking table serving as the alignment table, IR camera/table for detection of alignment marks, and a bonding head to load the pressure. The alignment procedures include three steps. The first two steps (i.e., image detection and coarse alignment) were accomplished with a sub-pixel image processing algorithm. After the coarse alignment, the misalignments were reduced within the moiré-based measurement range (about 1/4 pitch, ~5 μm in this study). Subsequently, step 3 (i.e., fine alignment) will be performed with the digital moiré fringes to align the two grating marks from 5 μm to tens of nanometer range.

**Figure 7:**
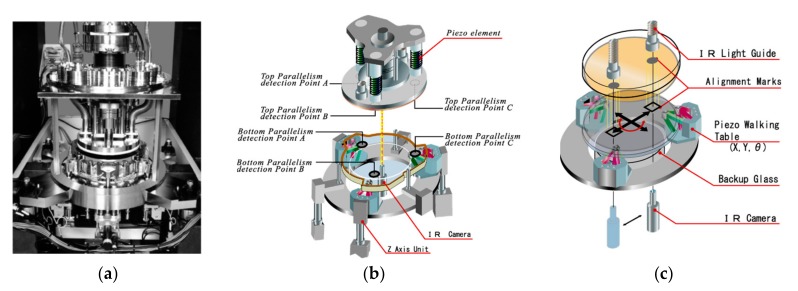
The aligned wafer bonding system used in this study: (**a**) photograph, the schematic diagrams of (**b**) the parallel adjustment and (**c**) the infrared (IR) alignment systems.

Figure 7a shows a photograph of the aligned wafer bonding system used in this study. The silicon wafer surfaces were activated with O_2_ reactive ion etching (RIE) plasma treatment for 30 s and followed by treatment with N_2_ radicals for 15 s. Afterwards, the top wafer was turned over and loaded into the aligned wafer bonding chamber on the wafer transfer pin, and then the bonding head was lowered together with the top electrostatic chuck.

The top wafer was held by charging the top electrostatic chuck and placed on the upper position. The activated bottom wafer was transferred into the chamber and placed on the bottom electrostatic chuck, which was charged to hold the bottom wafer. Next, three *z*-axis units attached to the piezoelectric walking table behaved as actuators for the parallel adjustment of the bottom wafer. The parallelism of the top and bottom wafers was calibrated through the height displacement of parallelism detection points A to C using lower camera and was automatically adjusted through the operation of the top and bottom actuators respectively (see Figure 7b). After that, the top and bottom wafers were set very close to each other (< 50 μm gap), and the alignment grating marks were captured by an IR camera with a 5× magnification objective lens, as shown in Figure 7c. In the coarse and fine alignment, the misalignments with Δ*x*, Δ*y*, and Δ*δ* were measured simultaneously. After the computer received the misalignment feedback, the bottom wafer was precisely aligned to the top wafer by adjusting the piezoelectric walking table. When the coarse and fine alignments were accomplished, the two wafers were bonded at room temperature (~25 °C).

**Figure 8:**
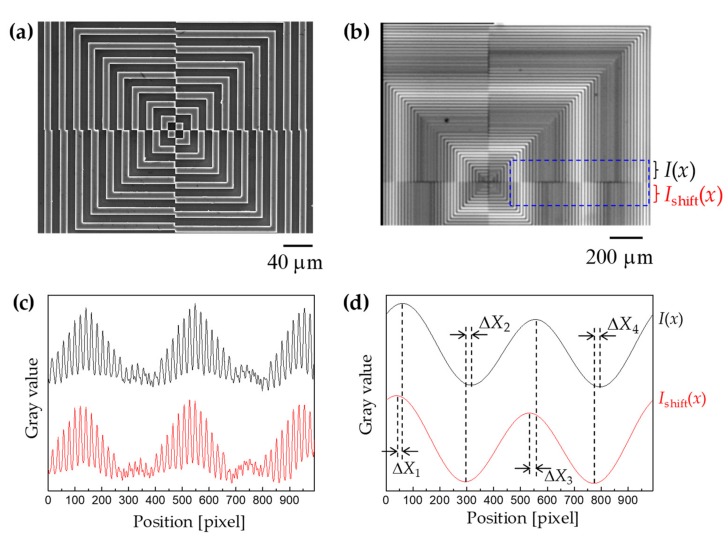
(**a**) Scanning electron microscope (SEM) image of the centrosymmetric grating mark fabricated on the Si wafer; (**b**) IR image of the moiré fringe pattern for the Si/Si wafer bonding; (**c**) the intensity distribution as a function of position along *x*-direction in the blue rectangular region; (**d**) the moiré shift in *x*-direction (Δ*X*, the average value of Δ*X*_1_*~*Δ*X*_4_*)* was measured by analysis of the moiré fringes after fast Fourier transform (FFT) and low pass filter.

Figure 8a shows the scanning electron microscope (SEM) image of the centrosymmetric grating mark fabricated on the Si wafer. The fabrication process was described previously in the literature [18]. In the coarse and fine alignments, the two superimposed grating images can be captured by the IR camera because the Si wafer is IR permeable. The IR transmission image of the moiré fringe pattern for the Si/Si wafer bonding is shown in Figure 8b. The two-dimensional digital image information was decomposed into two one-dimensional signals by averaging gray values along *x* or *y* directions, respectively. For example, the intensity distribution was as a function of position along the *x*-direction in the blue rectangular region of Figure 8c. Afterwards, the fast Fourier transform (FFT) was employed to analyze the frequency terms of the gray values, and a low pass filter was used to filter out the high frequency terms. Figure 8d shows a sinusoidal envelop curve, which was derived from the gray values. The peak-and-valley can address the position on *x*-axis more clearly. The moiré shift in *x*-direction (Δ*X*, the average value of Δ*X*_1_*~*Δ*X*_4_*)* was measured and the actual misalignment Δ*x* was subsequently determined by multiplying a factor of 1/*M* (1/40 in our case).

**Figure 9:**
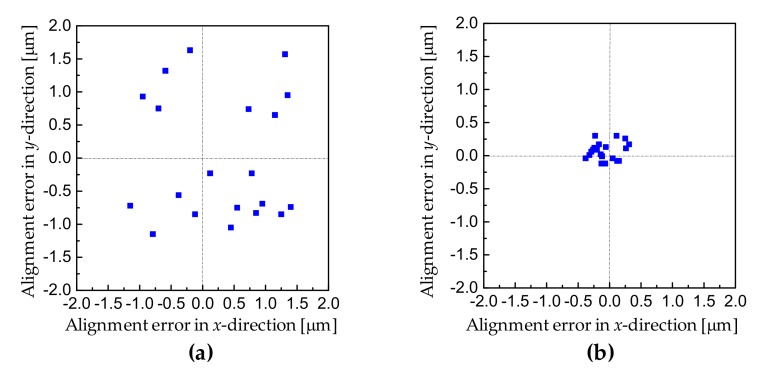
The alignment errors in *x*- and *y*- directions after bonding: (**a**) alignment using the cross-and-box marks; (**b**) alignment using the moiré-based centrosymmetric grating marks.

We tested the moiré-based alignment accuracy after the bonding. The alignment errors in *x*- and *y*- directions after bonding were measured and plotted in Figure 9. As a comparative study, the cross-and-box marks were also pre-fabricated on 4 inch Si wafers together with the centrosymmetric square marks. Firstly, the alignment was carried out using the conventional cross-and-box marks based on sub-pixel peak estimation algorithms. The alignment errors ((Δ*x*^2^ + Δ*y*^2^)^1/2^) were measured over twenty bonded pairs to get the standard deviation. The alignment accuracy for the bonded wafer was 1.20 μm (3σ). Secondly, the alignment was performed using the moiré-based centrosymmetric grating marks. The alignment accuracy was better than 300 nm (3σ), which was much smaller than in the former case. 

Compared to the case using the conventional alignment marks, the accuracy was improved by four times. Besides the alignment marks, the accuracy of wafer-to-wafer alignment was also determined by several factors, such as the resolution of microscopy, the positioning stage, and the parallelisms of wafers. In one study [8], the EV Group achieved the alignment accuracy of 200 nm using conventional alignment marks (i.e., standard keys) alone. This implies that their alignment tool exhibits superior performance compared with our tool (~1.2 μm merely). Therefore, we believe the alignment accuracy using moiré-based grating marks could be improved further when a high-precision alignment tool is employed. Moreover, according to Equation (5), a larger magnification factor (i.e., *M*) could increase the measurement accuracy further. For current status, the theoretical limit of the minimum resolved misalignment using moiré gratings is ~0.2 nm described in the literature [19]. This has great potential to get significantly higher alignment accuracy by optimizing the moiré grating parameters (i.e., *p*_1_, *p*_2_, and *P*).

**Figure 10:**
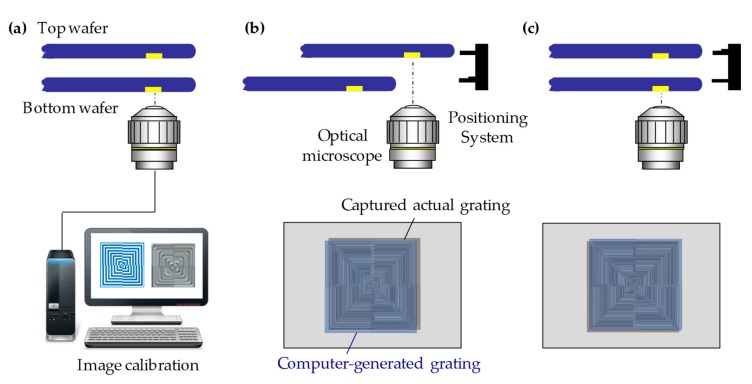
The working principle of moiré-based alignment for the backside alignment system: (**a**) the calibration between the computer-generated and the captured actual images; (**b**) detecting the top grating mark positions; (**c**) detecting the bottom grating mark positions and aligning two wafers by restoring the top and bottom mark positions.

On the other hand, the direct IR alignment method is not applicable for IR opaque wafers, such as high dosed silicon and large-scale integration (LSI). To overcome the problem, commercially available wafer-to-wafer bonding tools are often employed alignment system with backside alignment mark on top wafer with captured top-side alignment marks image on bottom wafer. So, we propose the working principle of the moiré-based alignment for the backside alignment system, as shown in Figure 10. The moiré grating marks were pre-fabricated on the backside of the bottom wafer. Meanwhile, the computer can also generate a digital moiré grating on the basis of the layout. An optical microscope (e.g., 20× magnification objective lens) was used first to capture the actual moiré grating images on the top and bottom wafers. The computer-generated grating image was calibrated with the captured actual images, for example, the grating size and the gray level, as shown in Figure 10a. As a second step, the optical microscope was used to detect the grating mark positions on the top wafer (Figure 10b). The computer-generated grating image was superimposed with the actual image captured and the moiré fringes were formed. The blurred fringes could be acquired by the fast Fourier transform (FTT) and the low pass filter process. Accordingly, the accurate positions of the top grating marks were recorded in the software. In the third step, the bottom stage was retracted for detecting the grating marks on the bottom wafer (Figure 10c). The accurate position was also addressed in terms of the moiré-based principle. The software was utilized to calculate the perfect overlay position for top and bottom wafers. The two wafers were finally aligned by restoring the positions of the top and bottom stages. 

## 4. Conclusions

In this study, we developed a two-dimensional moiré pattern superimposed by centrosymmetric grating marks. Using digital image processing, both small and large misalignments could be detected. Therefore, a moiré-based alignment method using centrosymmetric grating images was realized without requiring additional cross-and-box alignment marks. The misalignments could be measured with more accurately and conveniently. Combined with the aligned wafer bonding system, sub-300-nm alignment accuracy (3σ) was achieved after bonding. This accuracy can be further improved if a larger magnification factor of the moiré grating is adopted. Furthermore, the working principle of the moiré-based alignment for the backside alignment system was proposed to overcome the difficulty in bonding of opaque wafers. We believe that this method has great potential in 3D heterogeneous integration with accurate alignment needs.
